# Feasibility of the Internet Attachment–Based Compassion Therapy in the General Population: Protocol for an Open-Label Uncontrolled Pilot Trial

**DOI:** 10.2196/16717

**Published:** 2020-08-14

**Authors:** Daniel Campos, Mayte Navarro-Gil, Paola Herrera-Mercadal, Laura Martínez-García, Ausiàs Cebolla, Luis Borao, Yolanda López-Del-Hoyo, Diana Castilla, Eva del Río, Javier García-Campayo, Soledad Quero

**Affiliations:** 1 Department of Basic and Clinical Psychology and Psychobiology, Universitat Jaume I Castellón de la plana Spain; 2 Instituto de Investigación Sanitaria Aragón (IISAragon) Zaragoza Spain; 3 Department of Psychology and Sociology University of Zaragoza Zaragoza Spain; 4 Department of Psychology and Sociology, University of Zaragoza Huesca Spain; 5 Department of Personality, Evaluation and Psychological Treatment, Universitat de València Valencia Spain; 6 CIBER of Physiopathology of Obesity and Nutrition CIBERobn Madrid Spain; 7 Primary Care Prevention and Health Promotion Research Network, RedIAPP Madrid Spain; 8 Department of Psychiatry Miguel Servet Hospital Zaragoza Spain

**Keywords:** compassion, self-criticism, feasibility studies, internet, happiness, meditation

## Abstract

**Background:**

Compassion-based interventions delivered over the internet are showing promising results for the promotion of psychological health and well-being. Several studies have highlighted their feasibility, acceptance, and preliminary efficacy. However, this is an incipient field of research, and to the best of our knowledge, there are no data available from Spanish-speaking countries.

**Objective:**

The aim of this study is to investigate the feasibility, acceptance, and preliminary efficacy of the Internet Attachment–Based Compassion Therapy (iABCT), a web-based version of the Attachment-Based Compassion Therapy, in Spanish speakers from the general population.

**Methods:**

This feasibility study features a single-arm, uncontrolled, within-group design with an embedded qualitative and quantitative process evaluation at baseline, immediately after the intervention and at the 3-month follow-up. A minimum of 35 participants from the general population will be allocated to iABCT. Feasibility measures will include attrition rate, patterns of use of the web-based system, and participants’ acceptability, usability, and opinion. The primary outcome was measured using the Pemberton Happiness Index. Secondary outcomes were measured using the Compassion Scale, Self-Compassion Scale, Forms of Self-Criticizing/Attacking and Self-Reassuring Scale-Short form, Five Facets of Mindfulness Questionnaire, Relationships Questionnaire, General Health Questionnaire, Non-Attachment Scale, International Positive and Negative Affect Schedule Short Form, Purpose-In-Life Test, and difficulties regarding the practice of compassion (Compassion Practice Quality Questionnaire). Mixed models will be used to evaluate primary and secondary outcome measures. A qualitative content analysis of the participants’ qualitative responses will also be performed.

**Results:**

Enrollment started in February 2020 and will be finished in April 2020. Data analysis will start in October 2020.

**Conclusions:**

To our knowledge, this study will, for the first time, show data on the feasibility, acceptability, and preliminary efficacy of web-based compassion (and self-compassion) training—that is, the adapted iABCT—in Spanish speakers from the general population. Further aspects of their implementation (ie, facilitators, barriers, and unwanted effects) and mechanisms of change will be investigated. This study will allow the revision and fine-tuning of the developed intervention, study design, and planning procedures, as well as the initiation of a future randomized controlled trial.

**Trial Registration:**

Clinicaltrials.gov: NCT03918746. Registered on April 17, 2019. Protocol version 1, 6 March 2019.

**International Registered Report Identifier (IRRID):**

PRR1-10.2196/16717

## Introduction

### Compassion-Based Interventions

Compassion-based interventions (CBIs) focusing on cultivating compassion and self-compassion have recently been developed with promising results for the general population as well as for the treatment of a number of different psychological disorders [1-5]. In particular, *self-compassion*, which involves the directing of compassion toward oneself, is emerging as a strong predictor of well-being, psychological health, and quality of life [6-10]. A recent meta-analysis study of randomized controlled trials (RCTs), for the clinical and nonclinical population, showed significant between-group differences with moderate effect sizes with regard to cultivating compassion (*d*=0.55; *k* [number of studies]=4; 95% CI 0.33-0.78), self-compassion (*d*=0.70; *k*=13; 95% CI 0.59-0.87), and mindfulness (*d*=0.54; *k*=6; 95% CI 0.38-0.71), reducing depression (*d*=0.64; *k*=9; 95% CI 0.45-0.82), anxiety (*d*=0.49; *k*=9; 95% CI 0.30-0.68), and psychological stress (*d*=0.47; *k*=14; 95% CI 0.19-0.56) and improving satisfaction with life and well-being (*d*=0.51; *k*=8; 95% CI 0.30-0.63), which remained when active control comparisons were included [4]. Kirby [3] found 8 face-to-face established CBIs with 6 of them having RCTs and meta-analysis evidence [4]. Those 6 CBIs were Compassion-Focused Therapy [11], Mindful Self-Compassion [12,13], Compassion Cultivation Training [14,15], Cognitively Based Compassion Training [16-18], Cultivating Emotional Balance [19,20], and Compassion and Loving-Kindness Meditations [21,22]. The other 2 CBIs pending the publication of evidence were the ReSource Training Protocol [23] and the Being with Dying Programme [24]. Another systematic review and meta-analysis investigated the effectiveness of self-compassion–related therapies (ie, CBIs, mindfulness-based cognitive therapy, and acceptance and commitment therapy). It found greater improvements in promoting self-compassion (g=0.52; 95% CI 0.32-0.71) and in reducing psychopathology (depression: g=0.40, 95% CI 0.23-0.57; anxiety: g=0.46, 95% CI 0.25-0.66) in clinical and subclinical populations, although the results did not remain when analyses were restricted to the comparison between self-compassion–related therapies and active control conditions [2].

### Attachment-Based Compassion Therapy

To the best of our knowledge, the attachment-based compassion therapy (ABCT) program is the first CBI to have been originally developed and validated in the Spanish language [6]. To date, ABCT—in the face-to-face group format, specifically—has shown its efficacy and applicability in a nonrandomized controlled study of healthy people (ie, adults not having a psychological disorder and not receiving any psychiatric treatment) [1] and in an RCT for the treatment of fibromyalgia [5]. In a study on the general population, Navarro-Gil et al [1] found significant improvements in self-reported measures of self-compassion, dispositional mindfulness, and secure attachment style, as well as significant reductions in psychological disturbance, experiential avoidance, and levels of anxiety and avoidance toward social relationships as compared with the waiting list control group. Moreover, the authors pointed out that the significant increments in secure attachment style in the ABCT group were mediated by changes in self-compassion. The results of the RCT on patients with fibromyalgia revealed greater improvements in general health status, clinical severity, anxiety, depression, quality of life, and psychological flexibility in the ABCT group (combined with treatment as usual [TAU]) compared with an active control group (relaxation combined with TAU) at postintervention and 3-month follow-up. An interesting finding was that the effect sizes found were larger than those obtained when treating fibromyalgia using cognitive behavioral therapy (CBT) or mindfulness-based interventions (MBIs). Moreover, psychological flexibility partially mediated the relationships between the experimental condition (ABCT and TAU vs relaxation and TAU) and overall health status, pain catastrophizing, anxiety, and depression at follow-up. Nevertheless, these studies did not include any specific well-being outcomes, indicating that further research is needed in this regard.

### Internet-Delivered Compassion-Based Interventions

There are promising results that suggest the potential utilities of web-based compassion training. One of the first studies is the research conducted by Krieger et al [25], testing the feasibility of a web-based version of the Mindfulness-Based Compassionate Living program in self-referred participants suffering from harsh self-criticism (N=39). Authors found high levels of satisfaction with the program; significant increases in self-compassion, mindfulness, reassuring self, and satisfaction with life; and significant reductions in inadequate self, hated self, perceived stress, and fear of self-compassion from pretreatment to posttreatment and from posttreatment to follow-up at 6 weeks, with medium-to-large within-effect sizes (*d*=0.50-1.50). A replicated RCT in 122 participants supported the preliminary evidence, including the maintenance of the effects at 6-month follow-up. The dropout rate was 11.6%. Other adherence outcomes (ie, number of completed modules and self-reported number of completed exercises per week) predicted postintervention scores for self-compassion but not for depressive, anxiety, and distress symptoms in the intervention group.

Finlay-Jones et al [26] gathered preliminary data on the feasibility and effectiveness of the Internet-Based Self-Compassion Cultivation Program for Psychology Trainees (N=37) in a pilot study. After completing the 6-module program, participants reported significant changes in self-compassion, happiness, depression, perceived stress, and emotion regulation difficulties at postintervention and 3-month follow-up. Program feedback from participants revealed high average levels across modules for enjoyableness, relevance, comprehension, and learning and low-to-moderate average levels for difficulty. Recently, Eriksson et al [8] pointed out the effects of a brief web-based mindful self-compassion program on stress and burnout symptoms in a group of practicing psychologists (N=81) and the relationships between changes in self-compassion and self-coldness and changes in stress and burnout symptoms in an RCT. After the 6-week training (15 min per day of an online exercise), the results showed significant increases in self-reported self-compassion and reduced burnout symptoms, perceived stress (*d*=0.59), and self-coldness levels. Measures of distress were strongly related to self-coldness rather than self-compassion.

In summary, there is incipient evidence that supports the feasibility, acceptance, and preliminary effectiveness of CBIs delivered over the internet. However, to our knowledge, the existing research has mostly been conducted in English-speaking countries. Data regarding how Spanish speakers would accept CBIs are needed to determine the feasibility to apply it in this population, to cover this gap, and explore the potentialities and advantages of delivering compassion via the internet to promote personal resources and well-being beyond geographical barriers. According to *the Mental Capital and Wellbeing: Making the most of ourselves in the 21st century* project, it is expected that achieving small changes in overall well-being levels of the general population would consequently lead to higher decreases in percentages of mental health illness and subclinical disorders [27]. Thus, emphasizing positive psychological states, such as compassion, would act as protective factors for social, physical, and mental health. Specifically, Neff and Costigan [28] pointed out that treating oneself with care and compassion is a powerful way to enhance intrapersonal and interpersonal well-being.

In this regard, although the ABCT protocol was developed to be applied in Spanish speakers, there are no data available on its effects on the well-being outcomes of this population or on whether it could be delivered in a feasible way through the internet. Therefore, there is a need to continue exploring ways of approaching the general population with these effective resources, which is one of the main advantages of delivering self-applied interventions over the internet.

### Aims and Hypotheses

The main aim of this study is (1) to investigate the feasibility of the Internet Attachment–Based Compassion Therapy (iABCT), a web-based version of ABCT, in the general population. Additional objectives are (2) to analyze the preliminary effects of iABCT on happiness, compassion, self-compassion, dispositional mindfulness, general health, purpose in life, nonattachment, attachment styles, self-criticism, and positive and negative affects at postintervention and 3-month follow-up; (3) to explore cost-effectiveness by means of changes in overall health status levels; (4) to investigate the mechanisms of change, predictors, and associations between outcomes; and (5) to assess adverse or unwanted effects and facilitators and barriers to the intervention received.

The principal hypothesis is that iABCT will be feasible and well accepted by participants in terms of attrition, expectations, satisfaction, usability, and opinion. Second, iABCT will show efficacy in promoting significant changes with moderate-to-large within-group effect sizes in self-reported measures of happiness, compassion, self-compassion, dispositional mindfulness, self-criticizing, nonattachment, purpose in life, attachment, and overall health status. We also hypothesize that gains will be maintained at 3-month follow-ups. Third, changes in overall health levels will demonstrate the cost-effectiveness of iABCT. Fourth, significant associations will be found between changes in compassion, self-compassion, dispositional mindfulness, general health, purpose in life, nonattachment, attachment styles, self-criticism, positive and negative affects, and happiness. Specifically, improvements in happiness scores and overall health status will be predicted by self-reported self-compassion. Increments in secure attachment style would mediate the relationship between self-compassion and happiness. Finally, low rates of adverse or unwanted effects are expected.

## Methods

### Study Design

This feasibility study and open trial features a single-arm, uncontrolled, within-group design with 3 measurement points at baseline (preintervention), immediately after the intervention (postbaseline), and 3-month follow-up with an embedded qualitative and quantitative assessment. Participants will be allocated to iABCT. The study was registered under Clinicaltrials.gov (NCT03918746) and will be conducted following the extension of the Consolidated Standards of Reporting Trials (CONSORT) statement for pilot and feasibility studies [29], the Consolidated Standards of Reporting Trials of Electronic and Mobile HEalth Applications and onLine TeleHealth guidelines [30], and the Standard Protocol Items: Recommendations for Interventional Trials (SPIRIT) guidelines (Multimedia Appendix 1) [31]. The SPIRIT checklist was used as a guide for reporting this study protocol (version 1; March 6, 2019).

### Eligibility Criteria

The sample will consist of healthy adults from the community. Inclusion criteria are participants should (1) be aged 18 years or older, (2) have adequate knowledge and an understanding of spoken and written Spanish, (3) have a computer with speakers and internet access in a secure setting (home or private office), (4) have an email account, and (5) be able to use a computer and browse the internet. Exclusion criteria are (1) a diagnosis of a mental disorder according to the Diagnostic and Statistical Manual for Mental Health Disorders-Version 5 (DSM-5) [32], (2) alcohol or other substance abuse or dependence, (3) receiving psychiatric or psychological treatment, (4) engaging in ongoing formal meditation training (eg, mindfulness or compassion intervention), (5) presence of heart disease, cardiorespiratory illness, or other severe medical condition, (6) history of epileptic crisis, and (7) unavailability to complete the internet intervention because of surgery or medical intervention.

The study will include participants with and without prior meditation experience. Meditation experience and frequency of meditation will be registered and considered in the data analysis.

### Sample Size

A minimum of 35 participants is considered sufficient to cover the aims of this feasibility study and to provide precise and efficient estimations of parameters (ie, means, standard deviations, effect size, and confidence intervals) for the powering of a larger RCT. Sample estimation is based on the recommended range in the literature (eg, 24 and 50) [33-35] and in line with existing feasibility studies in this field [25,26].

### Recruitment

Recruitment will be conducted online using professional and nonprofessional social media (ie, Facebook, LinkedIn, and Twitter), on the website where the internet intervention will be developed and hosted [36], and through advertisements in local newspapers and radios. The study will also be announced in the Master in Mindfulness program at the University of Zaragoza (Spain) and emailed to lists of contacts interested in meditation issues (eg, students on the master’s program and former students, and people subscribing to the newsletter published by the Mindfulness and Compassion Research Group [37]). In addition, posters will be placed in the following locations: Universitat Jaume I, the University of Valencia, the University of Zaragoza (Teruel, Huesca, and Zaragoza campuses), Miguel Servet Hospital (Zaragoza), and the Arrabal Health Centre (Zaragoza). All participants will access the study voluntarily, and no reimbursement for their participation will be provided in any case. The internet intervention aims to promote psychological well-being but not treat any mental disorders or medical conditions.

People who are interested in the study will be asked to contact the research team, and they will be scheduled for an admission telephone interview to screen for the inclusion and exclusion criteria—including a diagnostic telephone interview—and an explanation of the research terms (ie, study design, intervention length, and intervention rationale). Participants meeting the eligibility criteria and giving their informed consent will be allocated to the web-based intervention. A user account and password will be provided via email to each participant for individual use. Baseline assessment will be completed on the website and via a telephone interview before the intervention commences. Participants will be free to withdraw from the intervention or study at any time and without providing justification. In such cases, the research group will endeavor to contact them to ask for reasons and collect data regarding the feasibility of the internet-based intervention developed.

### Internet Attachment–Based Compassion Therapy

The intervention will consist of an internet-delivered version of ABCT [7]. ABCT is a compassion protocol based on the attachment theory and thus includes practices to raise awareness and/or address maladaptive aspects, where appropriate, of attachment styles developed with parents [6]. This process is taught as a form of both compassion and self-compassion to improve present-day interpersonal relationships and well-being in general [1]. ABCT consists of 8 group sessions, each of which has a 2-hour duration (1 session per week), including theory and both formal and informal compassion and self-compassion exercises and practices such as receiving and giving compassion to oneself, friends, unknown people, and people deemed to be problematic; identifying their own attachment style; and understanding how it influences their current interpersonal relationships, together with daily homework assignments that should take 15 to 20 min to complete [1,6].

**Figure 1:**
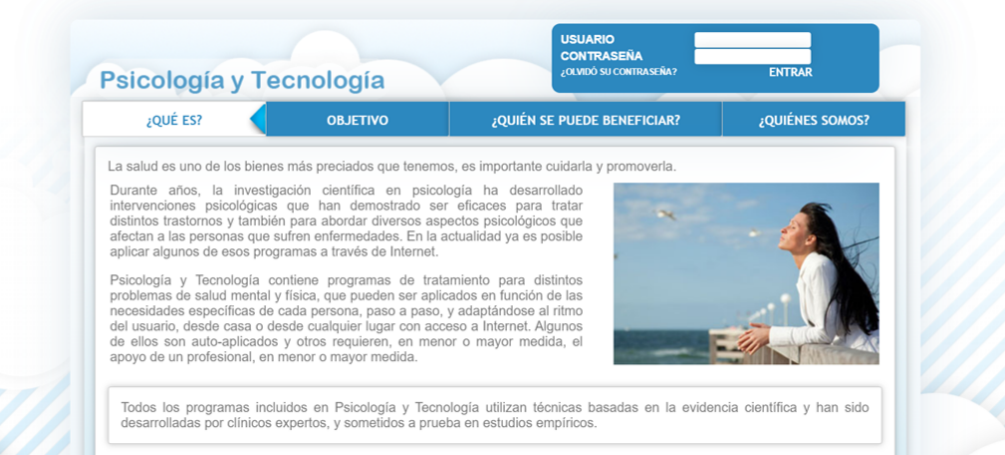
A screenshot of the “Psicología y Tecnología” (Psychology and Technology) Web platform.

iABCT will follow along the lines of the original model [6,7], and it will be adapted and developed to be totally self-applied over the internet via the website (Figure 1) [36], designed by the Laboratory of Psychology and Technology, Universitat Jaume I, and the University of Valencia. This platform allows several internet interventions including online assessments to be developed and hosted.

iABCT will consist of 8 sequential modules with the same structure: (1) module agenda; (2) theoretical contents of the module; (3) exercises and activities (including formal and informal practices) to put what is learned in the module into practice; (4) assessment of the knowledge acquired during the module; (5) tasks to be completed before advancing to the next module (homework assignments); and (6) summary of the module. The content will be presented through text, audios, videos, pictures, vignettes, and interactive exercises. Downloadable PDF files will be made available so that users can review them offline. Formal practices (guided meditations) will be delivered through audios with specific guides and instruction for each meditation. Furthermore, transcriptions of each guided meditation will be included as downloadable PDF files.

Table 1 presents the contents of each module. In comparison with the original ABCT, the adapted web-based version includes module 0, “Introduction to attachment-based compassion therapy,” to introduce to the participant the basics of the attachment-based compassion model and the program contents. Module 0 also includes tips about the use of formal and informal compassion (when/where/how much/how meditate) and on the importance of progressiveness in compassion training and of home practice between modules. Specific content has been added about “managing guilt” (formal meditation in module 4), “embarrassment” (theoretical component and formal practice in module 5), and “envy” (theoretical component and formal practice into module 7). Moreover, the term, “the figure of affect,” has been replaced by “basic affection” to make it easier to understand and so that participants will not confuse this with “the figure of secure attachment.” Other practices that have been eliminated are “showing forgiveness for the hurt caused by loved ones” (for people with grief experiences) and the “the illusion of labels” because they were not considered relevant for this protocol. Finally, module 8 on equanimity (“Beyond compassion: equanimity”) [6] has not been included in the web-based version because it was considered a different and advanced aspect of compassion.

The length of the interventions will depend on the pace of each participant, who will be advised to complete 1 module per week, taking the days between sessions to complete homework assignments. Each module has been optimized to allow it to be completed in approximately 1 hour. It is estimated that the web-based intervention can be completed in 8 weeks. However, each participant will be free to advance at his/her own pace with a maximum period of 10 weeks. Formal telephone support will not be systematically provided, but participants will be able to make contact for technical assistance (eg, web accessibility problems or forgotten passwords) if necessary.

**Table 1:** Structure and contents of Internet Attachment–Based Compassion Therapy.

Module	Theoretical component	Formal practice	Informal practice
0: Introduction to attachment-based compassion therapy	What is compassion? Contexts of application Attachment-based compassion therapy: structure and rationale Meditation and compassion: formal and informal practice Tips about meditation practice: when/where/how much/how meditate The importance of progressiveness in compassion and homework	N/A^a^	3-min compassionate practice
1: Preparing ourselves for compassion. Kind attention	The workings of our brain The reality of suffering: primary and secondary suffering What is and is not compassion?	Compassionate breathing and compassionate body scan Compassionate in coping with difficulties	Self-compassion diary Savoring and giving thanks
2: Discovering our compassionate world	Going deeper into compassion and mindfulness terms Compassion and related terms Fear of compassion	Connecting with basic affection Developing a safe place The compassionate gesture Identifying the figure of secure attachment	The object that joins us to the world Diary of compassion practice What are we good at?
3: Developing our compassionate world	How compassion works The figure of secure attachment Efficacy of compassion Self-criticism	Developing the figure of secure attachment Developing the compassionate voice	Writing a letter to the figure of secure attachment
4: Understanding our relationship with compassion	The biological bases of compassion Attachment styles Guilt Importance of these styles in everyday life	Becoming aware of our attachment style Ability to receive affection: friend, indifferent person, and enemy Guilty repair practice	Letter to your parents Observing our attachment styles in daily life
5: Working on ourselves	The importance of the affection toward ourselves and others Embarrassment	Showing affection to friends and indifferent people Showing affection to ourselves Reconciliation with our parents Repairing embarrassment	The greatest display of affection (in general and from our parents) 3 positive aspects and 3 negative aspects of our parents
6: Understanding the importance of forgiveness	The concept of forgiveness Phases of forgiveness Utility of forgiveness Basic resistances to generate forgiveness Resources to generate forgiveness The recapitulation (optional)	Forgiving yourself Asking others for forgiveness Forgiving others and showing compassion to enemies	Interdependence Compassion in daily life
7: Consolidating the practice of compassion	Working in 3 periods (past, present, and future) Envy Usefulness of being our attachment figure Difficult relationships How to keep up the practice of compassion for a lifetime	Working with envy Becoming our own attachment figure Handling difficult relationships	Not taking anything personally Our values and their relationship with compassion What would our lives be like if we started over?

^a^N/A: not applicable.

### Outcome Measures

Participants will be assessed at baseline (preintervention), postintervention, and 3-month follow-up after they complete the intervention. Assessments will be conducted online via the website [36], where the iABCT will be hosted, and via a telephone interview at pre- and postintervention, except for the 3-month follow-up that will be conducted only via the website. Both participants and researchers will receive email reminders of each assessment time. The study variables and assessment times are summarized in Table 2.

**Table 2:** Study measures, time of assessment, and source of measurement.

Measures	Aim	Assessment point	Assessment source
Admission interview	Screen eligibility criteria (inclusion/exclusion)	Pre^a^	Phone interview
Sociodemographic data	Sex, age, educational level, occupation, and civil status	Pre	Phone interview
M.I.N.I. 7.0.2.^b^	Psychological diagnosis (exclusion criterion)	Pre	Phone interview
Meditation data	Source of learning, frequency, duration of each session (in minutes), lifetime practice (in years), and context of practice	Pre	Phone interview
PHI^c^	Well-being	Pre, post^d^, and FW^e^	Web
SCS-26^f^	Self-compassion	Pre, post, and FW	Web
Compassion scale	Compassion	Pre, post, and FW	Web
FSCRS-SF^g^	Two forms of self-criticism: inadequate self and hated self and the ability to self-reassure	Pre, post, and FW	Web
FFMQ-15^h^	Dispositional mindfulness and mindfulness facets	Pre, post, and FW	Web
RQ^i^	Attachment styles	Pre, post, and FW	Web
GHQ-12^j^	Mental health recent symptoms, feelings, or behaviors	Pre, post, and FW	Web
NAS-7^k^	Nonattachment	Pre, post, and FW	Web
I-PANAS-10-SF^l^	Positive and negative affects	Pre, between modules, post, and FW	Web
PIL-10^m^	General sense of meaning and purpose in life	Pre, post, and FW	Web
Compassion Practice Quality Questionnaire	Difficulties related to the practice of compassion meditation	Between modules, post, and FW	Web
Expectations and Satisfaction Questionnaires	Participants’ expectations before the intervention and their satisfaction after it	Pre and post	Web
UAQ^n^	Usability and acceptability of the internet-based intervention	Post	Web
Qualitative opinion interview	Opinions on the internet intervention, the modules, and unwanted or unexpected effects	Post	Phone call
Attrition rate	Percentage of participants that drop out of the intervention after accessing it for the first time	Post	Web
Patterns of use for each participant	Length of the intervention, time spent on each module, and how many times they enter the modules	Between modules	Web

^a^Pre: preintervention.

^b^M.I.N.I. 7.0.2.: Mini International Neuropsychiatric Interview version 7.0.2 for DSM-5.

^c^PHI: Pemberton Happiness Index.

^d^Post: postintervention.

^e^FW: 3-month follow-up.

^f^SCS-26: Self-Compassion Scale.

^g^FSCRS-SF: Forms of Self-Criticizing/Attacking and Self-Reassuring Scale-Short form.

^h^FFMQ-15: Five Facets of Mindfulness Questionnaire.

^i^RQ: Relationships Questionnaire.

^j^GHQ-12: General Health Questionnaire.

^k^NAS-7: Non-Attachment Scale.

^l^I-PANAS-10-SF: International Positive and Negative Affect Schedule Short Form.

^m^PIL-10: Purpose-In-Life Test.

^n^UAQ: Usability and acceptability Questionnaire.

### Diagnosis, Screening, Sociodemographic, and Meditation Experience Data

An admission interview will be conducted to screen for inclusion and exclusion criteria. Sociodemographic data of participants will be recorded regarding sex, age, educational level, occupation, and civil status. Variables regarding overall meditation practice will be recorded as follows: any or no meditation experience, source of learning (ie, self-taught, therapy context, teacher or secular training course, and religious context), frequency of meditation (daily, 3 or 4 times a week, once a week or less, 2 or 3 times per month, sporadically, never), duration of each session (mean time in minutes), and lifetime practice (in years), and for participants with experience, the amount of time (in months) of meditation practice interruption and context of practice (secular or religious). Frequency, duration of each session (in minutes), and lifetime practice (in years) will also be asked for different meditation practice types (ie, focused attention meditation, open monitoring meditation, compassion or loving-kindness meditation, values mediation, deconstructive meditations, and informal mindfulness practices). A brief description of each meditation practice will be provided to guarantee the understanding and standardization of the concepts among participants.

Screening for exclusion criteria owing to the diagnosis of a psychological disorder will be performed using the *Mini International Neuropsychiatric Interview* (M.I.N.I. 7.0.2.) [38]. A copyright license for use of the standard M.I.N.I. 7.0.2 in Spanish, based on DSM-5 criteria, will be requested from the authors.

### Feasibility Outcomes

Feasibility outcomes will assess the implementation of iABCT regarding adherence (ie, attrition rate), patterns of use for each participant (eg, length of the intervention, time spent each in each module, how many times they enter the modules), participants’ acceptability (*Expectations and Satisfaction Questionnaires* adapted from Borkovec and Nau [39]), usability (*The Usability and Acceptability Questionnaire* [40,41]), and opinion (qualitative interview).

The *qualitative opinion interview* has been specifically developed to assess participant opinions on the web-based intervention. This semistructured telephone interview includes 14 questions with both quantitative and qualitative open questions: 7 of which refer to the usefulness of the intervention, components, modules, information provided, and multimedia elements (eg, images, audios, videos, PDF files) rated on a scale of 1 to 5 (1=not at all; 2=not very; 3=somewhat; 4=very; 5=extremely) and 2 dichotomous questions (“yes” or “no”) regarding whether they would consider it useful to have the program at their disposal for additional time after completion of the treatment, as well as whether they would like to have longer access. Additionally, interviewers would request to expand on the participants’ qualitative responses for each question. Finally, 2 open questions will be included to assess adverse or unwanted effects and facilitators and barriers to the intervention received.

### Psychological and Mental Health Outcomes

The primary outcome is well-being, which will be assessed using the *Pemberton Happiness Index* [42].

Secondary outcomes include the following: the *Compassion Scale* [43] and the *Self-Compassion Scale* [44] will be used to assess compassion and self-compassion, respectively. Self-criticism will be measured using the *Forms of Self-Criticizing/Attacking and Self-Reassuring Scale-Short form* [45]. Dispositional mindfulness will be assessed using the short *Five Facets of Mindfulness Questionnaire* [46]. The *Relationships Questionnaire* [47,48] will be used to assess attachment styles (ie, secure, preoccupied, dismissive, and fearful). The *General Health Questionnaire* (GHQ-12) [49,50] will be included to measure general mental health status. Nonattachment (eg, I can let go of regrets and feelings of dissatisfaction about the past when pleasant experiences end, I am fine moving on to what comes next) will be assessed using the *Non-Attachment Scale* [51], and positive and negative affects will be measured using the International Positive and Negative Affect Schedule Short Form [52]. The *Purpose-In-Life Test* [53] will assess the general sense of meaning and purpose in life. Difficulties related to the practice of compassion meditation will be assessed using the *Compassion Practice Quality Questionnaire* (adapted from Del Re et al [54]) that has been specifically developed for this study, which includes 10 items that participants score on a scale ranging between 0 and 100, indicating the percentage of the time that their experience reflects each statement.

### Ethics and Dissemination

This trial received approval from the Ethics Committee of Universitat Jaume I (Castellón, Spain; March 6, 2019; file number CD/006/2019) and will be conducted in compliance with the study protocol, the Declaration of Helsinki, and good clinical practice. Data security/confidentially will be guaranteed according to Spanish Organic Law 3/2018 of December 5 on the Protection of Personal Data and Guarantee of Digital Rights; all relevant EU and Spanish privacy laws will be observed and respected. Access will be granted to the internet platform via a unique username-password combination, and all transferred data will be secured using the advanced encryption standard polynomial *m*(*x*)=×8+×4+×3+×+1. Data collected via the website will be stored on secure servers at Universitat Jaume I, with personal data and user-generated data stored in separate databases on different servers. The consent form will be explained and required from all participants by researchers at the initial phone call. Written consent will be obtained before the start of the intervention.

A data monitoring committee (DMC) will be set up, comprising a psychiatrist, the principal investigator, and an independent clinical psychologist familiarized with the administration of internet-based interventions. The DMC will meet 3 times throughout the trial—after the baseline, posttreatment, and at follow-up measurements—but will be available on request at any time to provide support and information to all parties where necessary. The DMC will function independently of the sponsors and funders and will oversee and safeguard all trial participant interests, monitoring the overall conduct of the trial and ensuring the safety of participants by systematically checking negative events and reacting to any extreme distress or risk. In the case of an adverse event emergency, participants will be contacted and encouraged to receive additional help and counseling. Interim analyses are not contemplated in this study, although the DMC could request them if considered necessary for proper conducting of the trial and/or participant safety. Important protocol modifications will be communicated to relevant parties (ie, trial participants, trial registries, journals, ethical committee, and researchers). Results will be disseminated to relevant health care and professional communities and the general population via social media, in peer-reviewed and popular science journals, and at scientific and clinical conferences. Authors of the works derived from this study will be the investigators collaborating in this clinical trial, and there is no intention of using professional writers. A professional native English-speaking editor will check the language and grammar of English written content. Data generated in this trial (ie, full protocol, participant-level data set, and statistical code) will be made available upon reasonable request to the corresponding author.

### Patient and Public Involvement Statement

There was no involvement in the design and development of this trial by patients or the public. The public will be involved in the dissemination of the research. An end of study report will be developed to communicate study results to all participants, and a study newsletter will be sent to participants via email.

### Statistical Analysis Plan

Normality and homoscedasticity data assumptions will be checked using Kolmogorov-Smirnov (K-S) and Levene tests. Significant differences on categorical variables (eg, sex, educational level, occupation, etc) will be assessed using a chi-square test. Attrition and dropout rates will be calculated by reporting percentages and patterns of missing data. The Little missing completely at random (MCAR) test will be used to assess the assumption that data are MCAR [55]. Means and SDs will be reported for all the measures at each assessment point. A preliminary efficacy analysis will be performed using means of comparison for related samples based on intention-to-treat and per-protocol analyses. Mixed model analyses for primary and secondary outcome measures will be implemented using the linear mixed-effects models (MIXED) procedure with 1 random intercept per subject. *Time* will be treated as a within-group factor, and significant effects will be followed up with pairwise comparisons (adjusted by a Bonferroni correction). Sensitivity analyses will be performed to assess the robustness of the findings in terms of different methods for handling missing data (ie, mixed models with and without imputation, maximum-likelihood estimation, and maximum-likelihood multiple imputation) [56]. Within-group effect sizes (pre vs post and pre vs 3-month follow-up) will be reported using Cohen *d* and its 95% CI. For the analyses of associations between the intervention outcomes, predictors of change, and mechanisms of actions, several statistical tests will be performed such as Pearson correlation, multiple regressions, and mediation analysis using the bootstrapping approach [57]. A cost-effectiveness and cost-utility analysis will be conducted using the GHQ-12 based on literature proposals [58,59]. The mapping technique proposed by Lindkvist and Feldman [59] will be used to predict health state utility values using a crosswalk transformation algorithm from the GHQ-12 to the health utility measure (EuroQoL–5 dimension [EQ-5D]). Spanish tariffs of EQ-5D will be used as the reference population [58,60]. Additionally, participants’ qualitative responses regarding adverse or unwanted effects and facilitators and barriers to the intervention received will be explored using a qualitative content analysis and coding and the categorizing data approach by counting the frequency of words with NVivo software (QSR International). Statistical analyses of quantitative data will be performed using SPSS version 23 for Windows (IBM Corp). The statistical analysis plan (SAP) will be revised by all the study team members before the database is locked for use in the final statistical analysis. Any discrepancies or changes made between the analysis plan in this protocol and final SAP will be explained and reported.

## Results

Enrollment started in February 2020 and will be finished in April 2020. Data analysis will start in October 2020.

## Discussion

There is incipient evidence that supports the feasibility, acceptance, and preliminary effectiveness of CBIs delivered over the internet. However, to our knowledge, the existing research has mostly been conducted in English-speaking countries.

This paper describes the study protocol to investigate the feasibility of iABCT, a web-based version of the ABCT adapted to be completely self-applied over the internet for the general population. Results from this study will, for the first time, show data regarding the feasibility, acceptability, and preliminary evidence of web-based compassion (and self-compassion) training—that is, the adapted iABCT—in Spanish-speaking countries on a sample of healthy people. Moreover, further aspects of their implementation (ie, facilitators and barriers) and mechanisms of change will be investigated.

ABCT, in a face-to-face group format, has shown its efficacy and applicability for healthy people and for patients with fibromyalgia. This intervention focuses on training compassion and self-compassion to build a secure individual attachment style, which has been considered key for therapeutic efficacy [6,61]. It is expected that those improvements in compassion, self-compassion, and secure attachment will also promote an increased sense of well-being. In this study, specific measures of well-being and mental health (ie, happiness, overall health status, purpose in life, and positive and negative affects) have been included. Findings from this study will be congruent with the growing research supporting the benefits of using the internet to deliver evidence-based interventions [62-64] and will add valuable data to the incipient research field on the potential of self-applied CBIs via the internet [8,25,26,65].

We would like to highlight the relevance of conducting a feasibility study before designing a larger RCT due to the novelty of cultivating compassion via the internet (ie, CBIs). Moreover, despite the wide use of meditation-based interventions (eg, MBIs and yoga-based programs) over the last decade, compassion in the sense with which it is used here—the feeling that arises in witnessing another’s suffering and that motivates a subsequent desire to help [66]—is a relatively new concept for the general population in Spanish-speaking countries, where it is traditionally associated with a “feeling of commiseration and pity for those who suffer hardship or misfortune” [67]. However, the entry for this term in the official Spanish language dictionary of the Royal Spanish Academy has recently had its definition updated to a “feeling of pity, tenderness, and identification toward others’ afflictions” [68], which is closer to the psychological definition and the evolutionary perspective of compassion [66] and may reflect the assimilation of and re-conceptualization of the term.

This trial has been designed to cover the 8 areas of feasibility as suggested by Bowen et al [69]: *acceptability* (ie, how the intended individual recipients react to the intervention); *demand for the intervention* (ie, assessed by gathering data on estimated use or by actually documenting the use of a selected intervention); *implementation* (ie, the extent, likelihood, and manner in which an intervention can be fully implemented as planned and proposed, often in an uncontrolled design); *practicality* (ie, the extent to which an intervention can be delivered when resources, time, commitment, or some combination thereof is constrained in some way); *adaptation* (ie, to assess the necessity of changing program contents or procedures to be appropriate in a new situation); *integration* (ie, to assess the level of system change needed to integrate a new program or process into an existing infrastructure or program, as in our case using the website of Psychology and Technology); *expansion* (ie, to examine the potential success of an already-successful intervention with a different population or in a different setting, such as the original ABCT); *limited-efficacy testing* (ie, to test an intervention in a limited way, such as using a convenience sample, with shorter follow-up periods or with limited statistical power). In addition, this research will use a combination of methods (ie, qualitative and quantitative approaches) that best suit its feasibility study design based on author recommendations [70]. Key aspects of possible barriers to its implementation (ie, difficulties and unwanted or unexpected effects of the compassion meditation practice) will be directly asked and investigated.

Finally, based on recent literature findings, several constructs that have been associated with the promotion of well-being will be assessed together with compassion and self-compassion outcomes (eg, mindfulness, self-criticism, attachment styles, nonattachment, or purpose in life) [1,10,45,47,71-73]. Specifically, we would highlight the role of self-criticism (inadequate self and hated self and the ability to self-reassure), which is linked to various forms of psychological disorders [45,74]. Findings from this study would also provide preliminary data on associations between these variables.

## Appendix

Multimedia Appendix 1 Standard Protocol Items: Recommendations for Interventional Trials checklist.

## Abbreviations

ABCT: attachment-based compassion therapy
CBI: compassion-based intervention
CONSORT: Consolidated Standards of Reporting Trials
DMC: data monitoring committee
DSM-5: Diagnostic and Statistical Manual for Mental Health Disorders-Version 5
GHQ-12: The General Health Questionnaire
iABCT: Internet Attachment–Based Compassion Therapy
M.I.N.I: Mini International Neuropsychiatric Interview
RCT: randomized controlled trial
SAP: statistical analysis plan
SPIRIT: Standard Protocol Items: Recommendations for Interventional Trials
TAU: treatment as usual
